# The role of vascular endothelial growth factor-B in metabolic homoeostasis: current evidence

**DOI:** 10.1042/BSR20171089

**Published:** 2017-08-31

**Authors:** Mohammad Ishraq Zafar, Juan Zheng, Wen Kong, Xiaofeng Ye, Luoning Gou, Anita Regmi, Lu-Lu Chen

**Affiliations:** Department of Endocrinology, Union Hospital, Tongji Medical College, Huazhong University of Science and Technology, 1277 Jiefang Avenue, Wuhan 430022, China

**Keywords:** fatty acids, insulin resistance, metabolic syndrome, obesity, type 2 diabetes, VEGF-B

## Abstract

It has been shown that adipose tissue and skeletal muscles in lean individuals respond to meal-induced hyperinsulinemia by increase in perfusion, the effect not observed in patients with metabolic syndrome. In conditions of hyperglycaemia and hypertriglyceridemia, this insufficient vascularization leads to the liberation of reactive oxygen species (ROS), and disruption of nitric oxide (NO) synthesis and endothelial signalling responsible for the uptake of circulating fatty acids (FAs), whose accumulation in skeletal muscles and adipose tissue is widely associated with the impairment of insulin signalling. While the angiogenic role of VEGF-A and its increased circulating concentrations in obesity have been widely confirmed, the data related to the metabolic role of VEGF-B are diverse. However, recent discoveries indicate that this growth factor may be a promising therapeutic agent in patients with metabolic syndrome. Preclinical studies agree over two crucial metabolic effects of VEGF-B: (i) regulation of FAs uptake and (ii) regulation of tissue perfusion via activation of VEGF-A/vascular endothelial growth factor receptor (VEGFR) 2 (VEGFR2) pathway. While in some preclinical high-fat diet studies, VEGF-B overexpression reverted glucose intolerance and stimulated fat burning, in others it further promoted accumulation of lipids and lipotoxicity. Data from clinical studies point out the changes in circulating or tissue expression levels of VEGF-B in obese compared with lean patients. Potentially beneficial effects of VEGF-B, achieved through enhanced blood flow (increased availability of insulin and glucose uptake in target organs) and decreased FAs uptake (prevention of lipotoxicity and improved insulin signalling), and its safety for clinical use, remain to be clarified through future translational research.

## Introduction

The worldwide pandemic of obesity associated with net positive energy balance, sedentary lifestyle and increased consumption of animal fats, processed food and sugar has brought to forefront the need for novel therapeutic strategies to protect against the metabolic disturbances in such context [1]. A crucial, but poorly understood role of the endothelium is its ability to control the transport of energy supply according to organ needs [2]. Recent studies have identified crucial endothelial signalling mechanisms in charge of maintaining a balance of energy transfer and storage, and impairment of endothelial functions has been widely associated with metabolic alterations [3–5]. In turn, these alterations, including diabetes and insulin resistance (IR), lead to the abnormal behaviour of endothelial cells (ECs) [6]. It has been shown that obesity-related accumulation of neutral lipids in locations other than adipose tissue, especially in skeletal muscles in which most of the insulin-dependent disposal of glucose occurs, leads to IR by inhibiting the translocation of the glucose transporter 4 (GLUT4) from transport vesicles in the EC to its plasma membrane [7].

A large body of evidence suggests that the peripheral tissues (primarily skeletal muscle and subcutaneous adipose tissue) make the largest contributions to the clearance of orally ingested glucose and lipids, and thus make an important contribution to postprandial glucose and lipid homoeostasis [8–10]. In lean, healthy individuals, meal-induced hyperinsulinemia augments the resting blood volume of the skeletal muscle microvascular bed and this mechanism seems to be instrumental for glucose and lipid homoeostasis and long-term metabolic health [10,11]. Postprandial increases in perfusion, however, were not observed either in obese [12,13] and type 2 diabetes mellitus (T2DM) patients or in animal models of T2DM and IR [11,14,15]. Pathogenetically, adipose tissue inflammation, caused by insufficient vascularization and oxygenation, contributes to systemic IR and leads to disruption of nitric oxide (NO) synthesis [16], and exacerbation of the effects mediated by reactive oxygen species (ROS) [17]. This proinflammatory state disrupts the endothelial signalling mechanisms that mediate the normal uptake of circulating fatty acids (FAs), leading to their accumulation in non-adipose tissues such as the heart and skeletal muscles [2]. Excess FAs in these organs are widely associated with impairment of glucose uptake and insulin signalling, further promoting IR [18,19].

Members of vascular endothelial growth factor (VEGF) family, VEGF-A and VEGF-B, are involved in vascular inflammation and remodelling through increased proinflammatory and angiogenic mechanisms [20]. VEGF-A and VEGF-B use tyrosine kinase receptors: VEGF-A binds to VEGF receptor 1 (VEGFR1) and VEGF receptor 2 (VEGFR2), whereas VEGF-B binds to VEGFR1 exclusively [20,21]. A reciprocal regulation of adipogenesis and angiogenesis has been suggested, is an intense cross-talk between ECs of angiogenic vessels and preadipocytes a key determinant of both processes [22]. In that sense, it has been shown that the blockade of VEGF signalling by the inhibition of VEGFR tyrosine kinases impairs the development of adipose tissue in murine models of obesity [23–25]. Several authors have reported increased levels of circulating VEGF-A in human obesity [26,27]. Similarly, a significant decrease in VEGF-A concentrations was observed in patients with a dramatic weight loss following the gastric bypass, intensive dietetic intervention, and other bariatric surgery procedures [27,28]. Thus, it is becoming widely accepted that a perturbed cross-talk between adipocytes and ECs which takes place mostly through the VEGF/VEGFR system plays a key role in the pathogenesis of obesity and metabolic disturbances [29].

Because of its high sequence homology and similar receptor binding pattern to VEGF-A, VEGF-B was initially thought to be an angiogenic factor as well. However, the available evidence suggests that the role of VEGF-B in angiogenesis differs from one of other VEGFs [30]. Namely, it seems that the role of VEGF-B is multifaceted and context dependent: under degenerative conditions, VEGF-B inhibits the apoptosis of different types of vascular cells (ECs, pericytes and smooth muscle cells) to rescue the engendered blood vessels from degeneration, whereas in the presence of high levels of angiogenic/growth factors acts as an inhibitory factor, ensuring a balanced blood vessel density and tissue growth [30,31]. Some authors reported that VEGF-B may potentiate angiogenesis by increasing the bioavailability of VEGF-A [32], but the majority of them agree that VEGF-B cannot initiate angiogenesis or increase vascular permeability by itself [33–35]. In alignment with these observations is the fact that, opposite to VEGF-A, only one study found higher concentrations of VEGF-B in the serum of obese individuals [27]. However, as shown by several authors, endogenous VEGF-B levels are highest in tissues with high metabolic activity, such as the heart, skeletal muscle and brown adipose tissue [22,34], which implies an important metabolic role of this substance. Recently, the absence of VEGF-B was reported to lead to decreased expression of FA transport proteins (Fatp3 and Fatp4) in ECs, which correlated with decreased lipid droplets in cardiomyocytes and skeletal muscle fibres, and improved insulin sensitivity in diabetic models [4]. FAs represent a key energy source that is utilized by a number of tissues, but whose utilization must be tightly regulated to avoid potentially deleterious consequences of excess accumulation, including IR [3,36]. This review will summarize and discuss current preclinical and clinical evidence relevant for the role of VEGF-B in metabolic homoeostasis.

## Biology, tissue distribution and regulation of VEGF-B expression

VEGF-B is a growth factor encoded by the *Vegfb* gene, located on chromosome 11q13 in humans [37]. Due to alternative splicing, the *Vegfb* gene gives rise to two homodimers, VEGF-B167 and VEGF-B186, in both humans and mice. VEGF-B167 has a heparin-binding domain, so that upon secretion, VEGF-B167 binds to cell-surface heparin sulphate proteoglycans. By contrast, VEGF-B186 does not contain the heparin-binding domain and therefore is more soluble [37]. VEGF-B and its receptors are expressed by different types of vascular cells [30,33,34]. VEGF-B is expressed early during foetal development in mice, most readily in the heart, central nervous system (CNS) and brown adipose tissue [38–40]. In physiological conditions, it remains abundantly expressed in most tissues and organs in adult mice, especially in the cardiac myocytes, skeletal muscles and neuronal tissues [39]. In mice, VEGF-B167 is the predominant isoform expressed in most tissues and organs, accounting for more than 80% of the total VEGF-B transcripts, while VEGF-B186 is expressed at lower levels and in a limited number of tissues [39].

In physiological conditions in humans, VEGF-B is most abundant in tissues with high metabolic activity, such as the heart, skeletal muscle and brown adipose tissue [22,34], all of which are tissues enriched in mitochondria and which mainly use FAs as an energy source. Olofsson et al. [22] reported that VEGF-B transcripts were prominently expressed in the myocardium of an adult heart and adult striated muscle, but contrary to expected, no specific signal was detected in arterial smooth muscle. While the proportions of the expression of two different isoforms of VEGF-B in humans remain undefined, it has been shown that VEGF-B186 predominates in mice and human tumour cell lines [41]. *Vegfb* mRNA has been found in many different human tumour types: adenocarcinoma [42], breast [43] and ovarian carcinoma [44], lymphoma, melanoma, sarcoma [41] etc. Salven et al. [41] have shown that VEGF-B expression levels are higher in cancer tissue as compared with healthy tissue samples, suggesting its role in neoangiogenesis. On the other hand, in human ischaemic heart disease and dilated cardiomyopathy, Mehrotra et al. [2] reported a significantly decreased VEGF-B expression in both the diseases as compared with non-failing hearts.

### Factors that influence VEGF-B expression

To date, the molecular mechanisms responsible for regulation of VEGF-B expression remain poorly understood. Environmental stimuli such as hypoxia [45] and/or cold [46], which can induce VEGF-A expression do not seem to regulate levels of VEGF-B, due to lack of hypoxia-inducible factor-1 found in the *Vegfb* gene promoter [45]. Similarly, molecules that induce VEGF-A expression such as growth factors [45], prostaglandin E2 [47] or steroid hormones [43], do not appear to influence the expression of VEGF-B.

VEGF-B expression is high in mitochondria-dense tissues [22,34], and bioinformatic analysis showed that *Vegfb* is co-expressed with a set of nuclear-encoded mitochondrial genes, the so-called *OXPHOS* genes [3,48]. Trying to establish links between VEGF-B and known signalling pathways or metabolic networks, Hagberg et al. [3] found that VEGF-B expression was tightly co-regulated with a large cluster of nuclear genes coding for mitochondrial proteins across several mouse tissues. Specifically, VEGF-B was found to have a similar expression pattern as two mitochondrial markers—NADH dehydrogenase 1a subcomplex 5 (Ndufa5) and cytochrome *c* (Cycs) in response to different nutritional changes. For this reason, the authors postulated that VEGF-B expression might be regulated by the same factors as Ndufa5 and Cycs, especially by peroxisome proliferator activated receptor γ co-activator 1α (Ppargc1a/PGC-1α), a major regulator of mitochondrial energy metabolism. The co-expression of VEGF-B and mitochondrial proteins introduced a novel regulatory mechanism, whereby endothelial lipid uptake and mitochondrial lipid use are tightly co-ordinated [3]. Later on, the study of Mehlem et al. [49] demonstrated that *Vegfb* is indeed a downstream target of the PGC-1α/oestrogen-related receptor α (ERR-α) signalling pathway, as previously hypothesized by Hagberg et al. [3].

**Figure 1:**
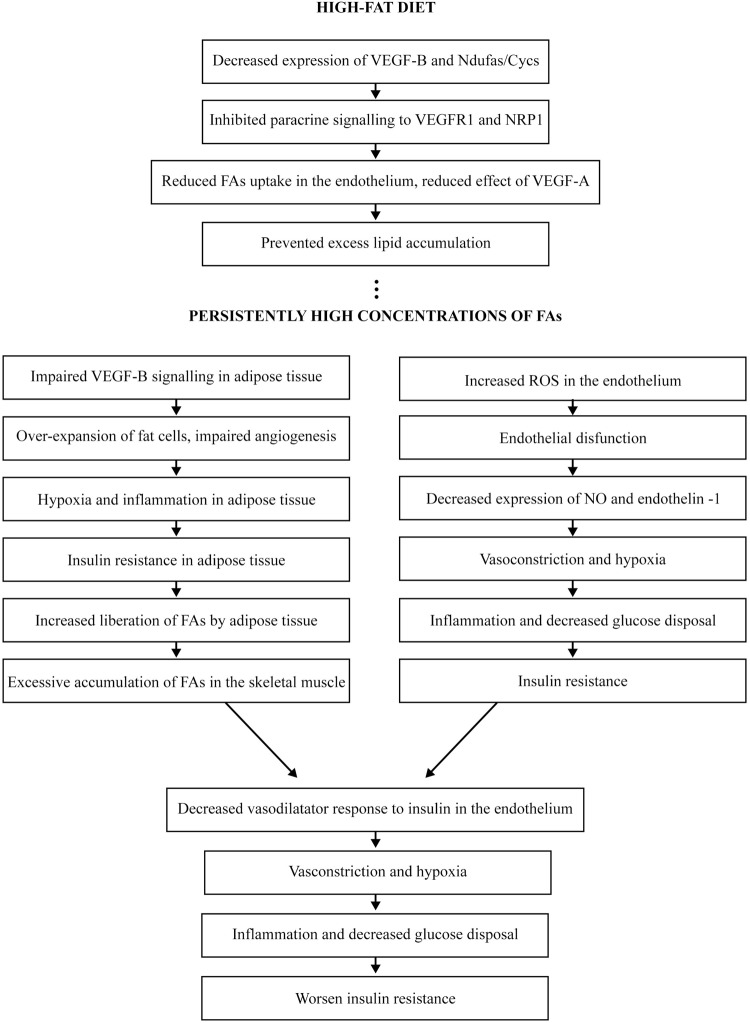
Hypothetic behaviour of VEGF-B in conditions of high serum concentrations of FAs and the subsequent chain of events leading to systemic IR Abbreviations: Ndufa, NADH dehydrogenase 1a subcomplex; NRP1, neuropilin 1.

PGC-1α is induced by physiological stimuli including exercise, fasting and cold temperature [49]. In response to extrinsic factors, PGC-1α binds to and co-activates several transcription factors, including Esrra/ERR-α, PPARγ and nuclear respiratory factor 1 (NRF1). In the normal state, PGC-1α co-ordinates mitochondrial biogenesis and β-oxidation with FA uptake and angiogenesis by co-expression of mitochondrial *OXPHOS* genes with *Vegfa* and *Vegfb* [49]. During nutrient deprivation and/or exercise, the expression of PGC-1α induces mitochondrial biogenesis that subsequently up-regulates expression of VEGF-B and FA uptake in the tissue cells, and induces expression of VEGF-A with subsequent angiogenesis. This type of regulation ensures that increased blood vessel growth and nutrient supply are correlated with the higher oxidative capacity in the tissue to preserve the metabolic homoeostasis [49]. In contrast, it would be logical to expect that in high-fat diets, PGC-1α-induced mitochondrial biogenesis and VEGF-B expression are inhibited, which lead to reduced FA uptake and decreased angiogenic and vasodilatory responses. The hypothetic behaviour of VEGF-B in conditions of high serum concentrations of FAs and the subsequent chain of events leading to systemic IR are presented in Figure 1.

## The role of endothelial dysfunction and VEGF-B in pathogenesis of IR

The effect of obesity to increase the risk of cardiovascular morbidity and mortality is strongly associated with IR in peripheral tissues such as the liver, skeletal muscle and adipose tissue [17]. Number of preclinical studies suggest that the pathogenesis of IR in models of both genetic (e.g. the Fatty Zucker rat) [37,38] and acquired (e.g. diet-induced) obesity [39] involves activation of some key transcriptional mediators of cellular inflammation in response to exposure to FAs or excess glucose, and insufficient vascularization and oxygenation.

As a primary regulator of carbohydrate, fat and protein metabolism, insulin plays a role as the key body protector against excess nutrient intake by using the adipose tissue, liver and skeletal muscle as biological buffers [16,17,36]. IR, an important feature of T2DM, obesity, glucose intolerance and dyslipidemia is also an important component of cardiovascular disorders, including hypertension (HTA), coronary artery disease and atherosclerosis, features also characterized by endothelial malfunction [16]. In turn, endothelial malfunction is present in T2DM, obesity and dyslipidemias [16]. In a proinflammatory milieu of excess FAs and/or glucose, the most obvious link between acquired IR and endothelial malfunction comprises lack of stimulation of the production of NO and endothelin-1 from endothelium caused by IR, with subsequent vasoconstriction, decreased blood flow and reduced glucose disposal in skeletal muscle which additionally worsens IR [17,40]. Thus, postprandial increases in perfusion that ensure a normal clearance of orally ingested glucose and lipid by the skeletal muscle and subcutaneous adipose tissue do not succeed and large transient increases in plasma glucose and triglycerides’ concentrations subsequently promote IR, T2DM and cardiovascular complications [41,42].

Therapeutic interventions in animal models and human studies have demonstrated that improving insulin sensitivity ameliorates endothelial dysfunction [40]. However, an opposite effect ameliorated IR as a result of an improved endothelial function has been demonstrated as well [40]. Seemingly, acquired IR and endothelial dysfunction share similar aetiopathogenetic factors: glucotoxicity in T2DM, lipotoxicity caused by elevated levels of FAs in T2DM, obesity and dyslipidemias, and proinflammatory states associated with metabolic and cardiovascular diseases [40]. All these factors are thought to underlie reciprocal relationships between IR and endothelial dysfunction and contribute to the linkage between metabolic and cardiovascular diseases [40].

VEGF-B seems to have a dual role in the pathogenesis of IR via regulation of endothelial FAs uptake and via modulation of tissue vascularity. The role of FAs is of particular importance in the pathogenesis of IR [36] and, as demonstrated recently, ECs play an active role in their homoeostasis [3]. As previously said, IR appears to be directly or indirectly related to diet-induced inflammation that interrupts insulin’s action by disrupting signalling mechanisms within the cell [17]. High levels of circulating FAs induce ROS production and impair endothelial function, whereas the overexpansion of existing fat cells creates hypoxia, which results in inflammation and subsequently IR in adipocytes [37]. With the development of cellular inflammation and IR in the adipocyte, higher levels of FAs can leave the fat cell to enter into the circulation and be at disposal to other organs, such as the liver and the skeletal muscles. Being unable to safely store large amounts of fat, these organs develop IR themselves [36]. The concept of endothelial mediated FA transport has existed for many years but has transitioned from a viewpoint of passive transfer to a highly regulated, active process that involves complex signalling pathways [1,3]. Recent studies have discovered a clear orchestration of endothelial FAs transport by VEGF-B and the molecules such as peroxisome proliferator activated receptor γ (PPAR-γ) and apelin, directly target the endothelium [1,3]. The absence of VEGF-B was reported to lead to decreased expression of FA transport proteins (Fatp3 and Fatp4) in ECs, which correlated with decreased lipid droplets in cardiomyocytes and skeletal muscle fibres [3], and improved insulin sensitivity in diabetic models [4]. Therefore, endothelium plays the role of a gate-keeper for uptake of FAs into skeletal muscle and as such may be a key factor for the distribution of lipids between skeletal muscle, subcutaneous adipose tissue and ectopic fat stores [1,3,4].

It is not clear whether regulation of FA uptake and modulation of tissue vascularity form part of one metabolic regulatory process or occur independently from each other. However, it has been shown that one of the key roles in the pathogenesis of obesity and metabolic disorders belongs to a perturbed cross-talk between adipocytes and ECs that as already mentioned, takes place mostly through the vascular VEGF/VEGFR system [22]. VEGF-A induction of angiogenesis in adipose tissue through enhanced vascularity, thermogenesis and a decrease in inflammation has been found to diminish metabolic complications caused by high-fat diet and the metabolic syndrome [50,51]. Accordingly, several authors have reported increased levels of VEGF-A in human obesity [26,27]. Similarly, a significant decrease in VEGF-A concentrations was observed in patients with a dramatic weight loss following the gastric bypass, intensive dietetic intervention and other bariatric surgery procedures [27,28]. It has been shown that this effect is mediated by VEGF-B that acts by increasing the bioavailability of VEGF-A and subsequent VEGFR2 activation [32]. Specifically, as demonstrated by Robciuc et al. [32], VEGF-B displays VEGF-A from its VEGFR1 receptor, which leads to activation of VEGF-A/VEGFR2 signalling and a cascade of events leading to improved insulin sensitivity and glucose tolerance. The role of VEGF-B in metabolic homoeostasis will be discussed in the upcoming chapters.

## Metabolic effects of VEGF-B: preclinical evidence

### FAs uptake regulation

As mentioned earlier, co-expression of *Vegfb* and mitochondrial genes ensures that endothelial uptake of FAs from the circulation is matched to the oxidative capacity of the tissue, thereby avoiding intracellular accumulation of excess lipids, subsequent lipotoxicity and IR [3,49]. As suggested by Hagberg et al. [3], VEGF-B produced by skeletal muscle fibres is released into the interstitium and diffuses into the abluminal membrane of ECs in the capillary EC layer (ECL). Here, it binds to VEGFR1 and neuropilin 1 (NRP1) which are both expressed by the ECL. Signalling of VEGF-B via a phosphatidylinositol-3-kinase (PI3K)-dependent mechanism then leads to the abundant expression of Fatp3 and Fatp4 in the ECL and incorporation into the luminal and abluminal membrane. This leads to increased *trans*-endothelial transport of FAs into the muscle interstitium where they diffuse to the muscle plasma membrane [3]. In the study of Hagberg et al., evaluation of *Vegfb^−/−^* mice showed that lipid uptake in mitochondria-dense tissues (heart, skeletal muscle and brown adipose tissue) was significantly decreased, while excess lipid uptake was observed in white adipose tissue with increased body weight of VEGF-B-deficient animals. This was associated with a likely compensatory increase in glucose utilization and insulin sensitivity, which was in part attributed to increased expression of GLUT4, a glucose transporter in heart and skeletal muscles [3]. Moreover, *Vegfb^−/−^* mice on either high-fat diet or on the *db/db* diabetic background (carrying mutation in the leptin receptor) were found to have normalization of glucose levels, improved β-cell function and ameliorated dyslipidemia [3,49]. The improved metabolic state was attributed to decreased lipid storage in the heart, skeletal muscle and pancreas, rendering these tissues to be more efficient in their glucose uptake. Furthermore, normalization of the high-density lipoprotein c (HDL-c) to low-density lipoprotein c (LDL-c) ratio, as well as decreased circulating levels of non-esterified FAs (NEFAs) and ketones were also seen in the *Vegfb*^*−/−*^mice [4]. The authors concluded that the vascular endothelium can function as an efficient barrier to excess muscle lipid uptake even under conditions of severe obesity and T2DM, and that this barrier can be maintained by inhibition of VEGF-B signalling [4]. In experimental mouse models of diabetic kidney disease (DKD), Falkevall et al. [52] showed that renal VEGF-B expression correlates with the severity of disease and that inhibiting VEGF-B signalling in DKD mouse models reduces renal lipotoxicity, resensitizes podocytes to insulin signalling, inhibits the development of DKD-associated pathologies and prevents renal dysfunction [52]. Similarly, Kivela et al. [53] found reduced FAs uptake in the heart and skeletal muscle of VEGF-B deficient rats, while Karpanen et al. [54] demonstrated that overexpression of VEGF-B in mice induced cardiac accumulation of ceramides, known for their implication in lipotoxicity and IR. However, other authors reported opposite findings. Robciuc et al. [32] found that *Vegfb* transduction reverted glucose intolerance in the preclinical mouse model of metabolic syndrome, stimulated fat burning, augmented basal oxygen consumption and metabolic rate, acting protectively against diet-induced obesity and metabolic complications. Nonetheless, when Robciuc et al. [32] experimentally limited VEGF-B expression to adipose tissue, they observed thermogenic effect similar to the one reported by Hagberg et al. [3]. Replication of high-fat diet studies in *Vegfb* gene-deleted mice [55], however, did not reproduce the findings of Hagberg et al. [3].

Contrary to expected, VEGF-B186, the more diffusible form of VEGF-B, was more effective than the heparin-binding form of VEGF-B, VEGF-B167, in inducing FA transport protein (FATP) expression in the study of Hagberg et al. [4]. The reason for this remains to be elucidated.

Supposing that Hagberg et al. [3] correctly hypothesized that VEGF-B produced by skeletal muscle leads to expression of endothelial Fatp3 and Fatp4, initiating transendothelial transport of FAs into the muscle fibres in high-fat diets and obesity, it would be reasonable to expect that decreased VEGF-B expression prevents lipid accumulation in skeletal muscle. This would subsequently lead to decreased effect of VEGF-A, that would impede further adipose tissue expansion. Indeed, preclinical evidence suggests that decreased expression of VEGF-B or inhibition of VEGF-B signalling pathways, not only prevents excess lipid storage in skeletal muscle, liver and adipose tissue but also leads to normalization of glucose levels, improved β-cell function and ameliorated dyslipidemia [4,52,53].

### Angiogenesis

Although some authors reported increased blood vessel permeability induced by VEGF-B186 [56], the scientific community generally agrees that neither VEGF-B167 nor VEGF-B186 have this effect [39,57]. VEGF-B seems to be inert under physiological conditions [39]. Nonetheless, as shown by both gain-of-function and loss-of-function analyses, both VEGF-B186 and VEGF-B167 are critically needed for blood vessel survival under pathological conditions [30,33,39].

Recently, Robciuc et al. [32] reported that *Vegfb* gene transduction into mice inhibits obesity-associated inflammation and improves metabolic health without changes in body weight or ectopic lipid deposition. The binding of VEGF-B to VEGFR1 activated the VEGF-A/VEGFR2 pathway and via different signalling pathways, including phospholipase C γ (PLCγ), PI3K/Akt and mitogen-activated protein kinase (MAPK), led to increased capillary density, tissue perfusion and insulin supply, signalling and function in adipose tissue. Endothelial *Vegfr1* gene deletion enhanced the effect of VEGF-B, activating the thermogenic programme in subcutaneous adipose tissue, which increased the basal metabolic rate, thus preventing diet-induced obesity and related metabolic complications. In obese and IR mice, *Vegfb* gene transfer, together with endothelial *Vegfr1* gene deletion, induced weight loss mitigated the metabolic complications, demonstrating the therapeutic potential of the VEGF-B/VEGFR1 pathway [32]. In 2014, Kivela et al. [53] showed that, by the same mechanism, VEGF-B dramatically expands the coronary arterial tree, increases functional coronary reserve, favours glucose oxidation and macromolecular biosynthesis in transgenic (cardiomyocyte-specific *Vegfb* transgene) rats. However, contrasting with a previous theory, VEGF-B expression did not influence FA uptake in transgenic, gene-targeted or wild-type rats [53].

**Figure 2:**
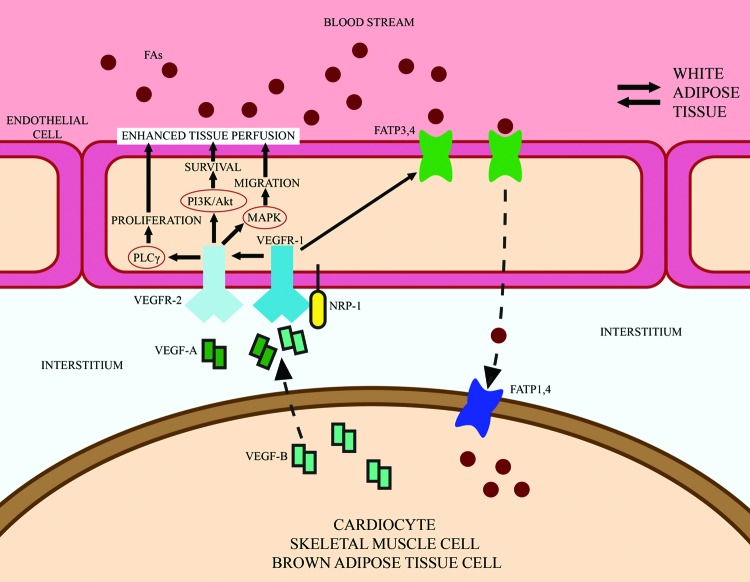
Schematic illustration on the role of VEGF-B in *trans*-endothelial FA transport and angiogenesis. VEGF-B secreted by cardiocytes, skeletal muscle cells and brown adipose tissue cells signals in a paracrine fashion to the receptors VEGFR1 and NRP1 located on the abluminal membrane of ECs. The binding of VEGF-B to VEGFR1 displays VEGF-A from its VEGFR1 receptor, activates the VEGF-A/VEGFR2 pathway and increases capillary density and tissue perfusion, while stimulation of ECs with VEGF-B up-regulates the expression of vascular FATPs and induces subsequent transport of FAs across the EC layer into tissue cells. Abbreviation: NRP-1, neuropilin 1. Modified from [67]: Hagberg C., Mehlem A., Falkevall A., Muhl L. and Eriksson U. (2013) Endothelial fatty acid transport: role of vascular endothelial growth factor B. *Physiology (Bethesda)*, **28** (2), 125–134)

It is still unknown how a VEGF-B-enhanced perfusion would lead to improved thermogenesis and insulin sensitivity in humans [32]. Increased insulin delivery as a consequence of increased vascularity might alleviate metabolic syndromes, but hypoxia markers as a formal proof of restored tissue oxygenation have not yet been investigated [32]. However, since metabolic events associated with obesity and IR occur in adipose tissue earlier than in any other tissue in humans, adipose tissue should be a starting point for researchers, especially in these very early stages of VEGF-B research. Figure 2 provides a schematic illustration of the role of VEGF-B in *trans*-endothelial FA transport and angiogenesis.

## Metabolic effects of VEGF-B: clinical evidence

In humans, a meta-analysis of genome-wide association studies showed a relationship between angiogenesis and IR, confirming that chronic ischaemia plays an important role in the latter, along with FAs [58]. However, although in animal models it has been shown the vascular endothelium can function as an efficient barrier to excess muscle lipid uptake even under conditions of severe obesity and T2DM by antagonizing VEGF-B signalling [2], clinical data related to the pathological roles of VEGF-B in obese patients and those with metabolic syndrome are very scarce.

In 2009, Gomez-Ambrosi et al. [27] compared serum concentrations of VEGFs in 15 lean and 24 obese patients. Circulating levels of VEGF-A, VEGF-B and VEGF-C were significantly increased in obese individuals, whereas levels of VEGF-D were significantly lower in this group as compared with lean individuals [27]. The same group previously reported increased expression of *Vegfb* gene in omentum of obese patients [59], thus higher serum concentrations of VEGF-B were in agreement with the previous results. However, increased VEGF-B concentrations in obesity were not confirmed by other authors. Aiming to establish the relationship between adipose tissue angiogenic capacity, obesity and IR, in 2012 Tinahones et al. [60] compared angiogenic factor expression levels in subcutaneous and omentum adipose tissues from morbidly obese patients with low (healthy obese) and high degrees of IR, and lean controls. They found that, while VEGF-A expression in both adipose tissues was up-regulated three-fold, expression of VEGF-B, VEGF-C and VEGF-D were decreased in both groups of obese patients as compared with lean patients, especially in those with high degrees of IR. The authors hypothesized that down-regulation of these angiogenic factors could be related to an alteration in the insulin sensitivity signalling pathway and that the enhancement of VEGF-A, could probably be in response to the impaired lymphangiogenic capacity, reflected by VEGF-B, VEGF-C and VEGF-D reduction [60]. Sun et al. [51] compared serum levels of VEGF-B in T2DM and healthy controls independent of their body mass index (BMI) and found no significant difference between the two groups. Serum VEGF-B levels in diabetic patients were significantly associated with the levels of c-peptide, total cholesterol and triglyceride but not with homoeostasis model assessment of IR, HDL or LDL. These findings led the authors to the conclusion that high serum VEGF-B levels might correlate with the presence of hyperlipidemia and target organ damage in T2DM patients [51].

In a group of 103 women with polycystic ovary syndrome and 96 age-matched healthy controls, Cheng et al. [61] found that both lean and overweight/obese patients with polycystic ovary syndrome had higher plasma VEGF-B levels than the healthy controls (*P*<0.05) and that VEGF-B levels were correlated with BMI, body fat percentage, homoeostasis model assessment of IR and β-cell function indices. Moreover, in patients with polycystic ovary syndrome treated with metformin, this drug treatment reduced VEGF-B levels and ameliorated IR [61].

So, growing scientific evidence indicates that angiogenesis is a process involved in adipose tissue expansion that takes place in obesity [50,51]. Suboptimal fat tissue perfusion can limit its expansion capacity and provoke inflammation, thus leading to systemic metabolic complications [16]. High circulating concentrations of VEGF-A found in obese patients are in accordance with this theory, as this factor is the key regulator of angiogenesis and vascular permeability [25]. If the binding of VEGF-B to VEGFR1 activates the VEGF-A/VEGFR2 pathway thus increasing capillary density, tissue perfusion and insulin supply and signalling by the mechanism proposed by Robciuc et al. [32], it would be to expect that circulating levels of VEGF-B in obese patients are increased as well. However, while Robciuc et al. [32] and Kivela et al. [53] showed that potentiation of VEGF-B effect by gene transduction or *Vegfbr1* deletion in animal models increased functional vasculature and mitigated metabolic disturbances, only two clinical studies found the positive correlation between VEGF-B serum concentrations and BMI [27,61]. Another two clinical studies reported different results when it comes to VEGF-B expression levels: while Tinahones et al. [60] found a significantly decreased VEGF-B expression in obese individuals with both low and high levels of IR compared with lean individuals, Sun et al. [51] found no correlation between BMI, levels of IR and the amount of VEGF-B.

On the other hand, if supposed that VEGF-B is one of the key regulators of FAs endothelial transport, and that a decreased VEGF-B expression is the first response to high amounts of circulating FAs, it would be interesting to know in what moment and how a persistent metabolic imbalance impairs VEGF-B signalling, leading to pathological lipid accumulation, overexpansion of fat cells, hypoxia, inflammation and subsequent IR. Is accumulation of ROS in endothelium a factor that takes a role in the endothelial dysfunction that precedes and possibly contributes to the development of IR? Is increased VEGF-B signalling responsible for pathological lipid accumulation and would in these pathological conditions inhibition of VEGF-B signalling have any effect? Or, is obesity in human potentially associated with some functional defect of VEGF-B? In the study where patients with the polycystic syndrome were compared with healthy individuals, the authors reported high levels of VEGF-B in both lean and obese patients, finding that VEGF-B correlates with BMI and the level of IR [61]. They reported that metformin reduced VEGF-B levels and ameliorated IR. However, knowing that metformin stimulates FAs oxidation with inhibition of cholesterol and triglyceride synthesis in the liver, promote FAs oxidation and glucose uptake in skeletal muscle and systemically increases insulin sensitivity itself, the question remains—does the regained metabolic balance reflex the effect of metformin, of decreased VEGF-B levels and subsequent impaired FAs uptake or something else?

## VEGF-B as a potential therapeutic agent for metabolic disorder and obesity

As mentioned earlier, the binding of VEGF-B to VEGFR1 activates the VEGF-A/VEGFR2 pathway leading to increased capillary density, tissue perfusion and insulin signalling and supply in adipose tissue in animal models [32]. Enhanced effect of VEGF-B achieved by gene transfer or *Vegfr1* gene deletion increases basal metabolic rate, induces weight loss and mitigates the metabolic complications in IR-obese mice [32]. The influence of down-regulation of VEGFR1 and increased VEGF-B levels on the thermogenic capacity of animal subcutaneous adipose tissue has been confirmed by other authors as well [62].

It is well established that adipose tissue inflammation is a major contributor to systemic IR in T2DM and obesity [63]. Suboptimal vascularization and perfusion of adipose tissue can limit its expansion capacity and provoke inflammation, thus leading to systemic metabolic complications [64]. The fact that VEGF-B increases perfusion and improves insulin delivery in obese adipose tissue could be important for the therapy of T2DM in two principal ways: (i) increased availability of insulin to target organs improves insulin sensitivity and (ii) insulin-induced capillary recruitment and increased blood flow facilitates glucose uptake in target organs [29]. Therefore, it is highly needed to elucidate the expression and functional status of VEGF-B in obese patients and those with metabolic syndrome, and to verify whether these conditions in humans may potentially be associated with any functional defect of VEGF-B. In their study on animal models of diabetic heart, Lal et al. [65] hypothesized that the immediate response to high glucose is the release of cardiomyocyte-surface bound VEGF-B, which triggers signalling pathways and gene expression to influence EC (autocrine action) and cardiomyocyte (paracrine effects) survival. They suggested that conditions of persistent hyperglycaemia eventually lead to impaired VEGF-B signalling in spite of an increase in VEGFR1 expression, inducing cell death [65].

In preclinical studies, recombinant VEGF-B increased functional coronary vasculature, reprogrammed cardiomyocyte metabolic pathways and protected the rat heart from ischaemic damage [53]. Nonetheless, in humans, low VEGF-B levels predict left ventricular remodelling after acute myocardial infarction (AMI) and VEGF-B expression is reduced in human cardiomyopathy [66]. It is still not clear whether overexpression of VEGF-B would induce favourable changes in the human coronary vasculature, cardiac function and myocardial metabolism, but since patients with T2DM suffer AMI more frequently and their outcomes are more severe than the healthy population, they could have a particular benefit from cardioprotective properties of VEGF-B.

In the study of Kivela et al. [53], high amounts of recombinant VEGF-B were very well tolerated by the treated animals. Potentially attractive safety profile of VEGF-B remains to be confirmed in clinical studies.

It is reasonable to expect that pathological lipid accumulation in obesity and metabolic syndrome may be affected by VEGF-B, considering its critical role in FAs uptake regulation [3]. However, besides some promising preclinical data [3,4,52,61], currently there is no confirmation of the preventive role of VEGF-B in pathological lipid accumulation and related metabolic complications in humans. FAs uptake affects numerous biological processes in the body, including cardiovascular, neurological and immune functions [40], and it is largely unknown how manipulations with VEGF-B would affect other biological functions. Moreover, the safety profile of VEGF-B for humans still remains unclear. Once the uncertainties regarding the divergent preclinical findings on VEGF-B’s role in metabolism are sorted out, a careful clinical approach should determine the clinical relevance of the endothelial signalling in the context of metabolic syndrome, thus providing greater insights that may ultimately lead to therapeutic advances against the increasing burden of the obesity pandemic and associated metabolic derangements.

## Perspectives

### Background

While the axes ‘impaired perfusion – inflammation – IR’ has been widely accepted as a basis of IR development in patients with metabolic syndrome, the exact signalling pathways responsible for it are still open to many questions. The angiogenic role of VEGF-B’s counterpart, VEGF-A, is clearly reflected through its increased circulating concentrations in obesity and metabolic syndrome, but VEGF-B seems to have a more complex role in metabolic homoeostasis.

### Results

Preclinical studies agree over two crucial metabolic effects of VEGF-B: (i) regulation of FAs uptake and (ii) regulation of tissue perfusion via activation of VEGF-A/VEGFR2 pathway. However, it is not clear whether these are two steps of the same process or are independent from each other. Very divergent preclinical evidence suggests that both VEGF-B antagonism and potentiation may have beneficial effects in preventing lipotoxicity and improving insulin sensitivity, but the mechanisms governing the functional switch of VEGF-B under different conditions are unknown. Currently, clinical evidence regarding levels of VEGF-B in circulation or adipose tissue in obese compared with lean patients is conflicting and scarce. Elucidating blood levels of this growth factor in healthy, non-obese patients, obese patients without metabolic syndrome and obese patients with metabolic syndrome may represent a promising first step towards better understanding of a complex VEGF-B’s role in humans.

### Potential therapeutic significance

Increased perfusion, improved insulin delivery and cardioprotective features of VEGF-B may be of particular benefit for patients with T2DM and ischaemic heart disease if future clinical and translational researches confirm these effects in humans and VEGF-B proves safe for human use. Hence, the expression and functional status of VEGF-B in obese patients and those with metabolic syndrome require the immediate attention of the scientific community.

## Abbreviations

AMI: acute myocardial infarction
BMI: body mass index
Cycs: cytochrome *c*
DKD: diabetic kidney disease
EC: endothelial cell
ECL: endothelial cell layer
ERR-α: oestrogen-related receptor α
FA: fatty acid
Fatp: fatty acid transport protein
GLUT4: glucose transporter 4
IR: insulin resistance
Ndufa5: NADH dehydrogenase 1a subcomplex 5
NO: nitric oxide
NRP1: neuropilin 1
PI3K: phosphatidylinositol-3-kinase
ROS: reactive oxygen species
T2DM: type 2 diabetes mellitus
VEGF: vascular endothelial growth factor
VEGFR: vascular endothelial growth factor receptor
